# Photoinduced and Classical Sol-Gel Synthesis: Spectral and Photophysical Behavior of Silica Matrix Doped by Novel Fluorescent Dye Based on Boron Difluoride Complex

**DOI:** 10.3390/polym13162743

**Published:** 2021-08-16

**Authors:** Jameelah Al-Harby, Haja Tar, Sadeq M. Al-Hazmy

**Affiliations:** Department of Chemistry, College of Science, Qassim University, Buraidah 51452, Saudi Arabia; Jhhrby@qu.edu.sa

**Keywords:** boron difluoride complex, sol gel glass, photosol-gel matrix, organic–inorganic matrix, TEOS, MMA, gold nanoparticles

## Abstract

The boron difluoride complex is known as an extraordinary class of fluorescent dyes, which has attracted research interest because of its excellent properties. This article reports the optical properties such as absorption, fluorescence, molar absorptivity, and photo-physical parameters like dipole moment, and oscillator strength of new fluorescent organic dye based on boron difluoride complex 2-(1-(difluoroboraneyl)-1,2-dihydroquinolin-2-yl)-2-(1-methylquinoxalin-2-ylidene) acetonitrile (DBDMA). The spectral characterization of the dye was measured in sol-gel glass, photosol-gel, and organic–inorganic matrices. The absorption and fluorescence properties of DBDMA in sol-gel glass matrices were compared with each other. Compared with the classical sol-gel, it was noticed that the photosol-gel matrix is the best one with immobilized DBDMA. In the latter, a large stokes shift was obtained (97 nm) and a high fluorescence quantum yield of 0.5. Special attention was paid to the addition of gold NPs into the hybrid material. The fluorescence emission intensity of the DBDMA with and without gold nanoparticles in different solid media is described, and that displayed organic–inorganic matrix behavior is the best host.

## 1. Introduction

Organic dyes are fluorescent molecules with an appropriate high molecular weight, characterized by containing extended systems of conjugated double bonds, i.e., separated by one bond at most (-C=C-C=C). Most of these molecules were considered as a laser dye [1], dissolved in an organic solvent or incorporated into a solid matrix. One of the promising areas is the production of dye-doped hybrid materials as functional materials for various practical applications [1,2]. The problems posed by liquid dye lasers stimulated a further consideration approach, and in the early 1990s the development of improved host materials with higher laser-damage resistance [3,4] and the synthesis of new high-performance laser dyes [5,6]. Lately, more effort has been devoted to incorporating organic laser dyes into various host matrices with the aim of developing solid state dye laser materials that could eventually replace liquid dye lasers [7,8,9]. The solid-state dye lasers have many advantages such as being compact, non-toxic, non-flammable, non-volatile, and better both mechanically and thermally [10]. Solid-state tunable dye laser materials can be developed by incorporating stable laser dye molecules into the solid matrices involving the use of new polymeric formulations, silica gels, xerogels, organically modified silicates (ORMOSILS), and glasses sol-gel as host materials for laser dyes that are competitive with their liquid counterparts [11,12].

Different families of organic dyes such as coumarin, rhodamine 6G, and BODIPY have been active components in hybrid materials and have a good response as dye lasers [13]. In recent years, research has continued to develop pirromethene-BF2 complexes (BODIPY) [14]. These complexes are an extraordinary class of fluorescent dyes that have aroused remarkable research interest due to their unusual and excellent properties. These properties include high fluorescence quantum yields, high molar extinction coefficient, spectra of precise emission, and exceptional chemical, thermal, and thermal photochemical stability [15].

Lately, Boron dipyrromethene complexes have attracted great interest from researchers, due to their interesting optical and chemical properties in organic media [16,17,18]. In the solid state BODIPYs, like most fluorophores undergoes fluorescence quenching, due to the tight close packing especially in the crystalline form, which leads to self-quenching [19]. For this reason, many strategic solves have been found to enhance solid state emission such as substitution in the following at the meso position and also in 2,6-positions and 3,5-positions result in more spaced packing in the solid state, resulting in highly fluorescence in solid materials (powders or films) [18,20]. Thus, studies of BODIPY obtained by means of the sol-gel process are still of continuing interest at present as it retains the properties of luminophores in hybrid materials, and the active ingredient becomes extra resistant to raised temperatures and strong ultraviolet radiation [21]. Moreover, generally low stokes shifts (<15 nm in most cases) are one of the significant drawbacks in the properties of BODIPY dyes [22] which causes self-quenching in the solid state [8]. In contrast, stokes shifts of >80 nm are favorable to reduce the re-absorption of emitted photons [23]. Furthermore, a wide variety of the BF_2_ complexes were reported in the literature with the appearance of some published scientific papers and some patents [20] which are structurally similar to BODIPYs but differ according to the aromatic rings surrounding the central hexagonal ring, unlike the five-member rings of BODIPYs [24]. While the dipyrrin ligand itself is non-emissive, the compounds are high-quantum-yield emitters when complexed to the -BF_2_ unit. The chromophore is largely associated with the rigidified dipyrrin ligand [23]. Recently, various kinds of functional nanoparticles have been developed and such as colloidal gold nanoparticles [25], and magnetite (Fe_3_O_4_) nanoparticles [26]. Fluorescent dye-doped nanoparticles have attracted special attention from researchers for several reasons such as that they can provide a high intensity of fluorescent for the measurement, and the exclusion of oxygen by matrix encapsulation [27]. The fluorescence intensity of a dye molecule is either quenched or enhanced in the close proximity of the metallic nanoparticles [28]. These advantages indicated that dye molecules encapsulated in the nanoparticles had high stability and retained their optical activity, thus providing a viable route for various applications with unique properties such as biochemical and bioanalytical applications and negating the need for additional reagents or signal amplification steps [27,29].

This study aimed to perform investigations on the spectral and photophysical characteristics of 2-(1-(difluoroboraneyl)-1,2-dihydroquinolin-2-yl)-2-(1 methylquinoxalin-2-ylidene) acetonitrile, new fluorescent organic dye (DBDMA) in different media (liquid, sol-gel glass, and photosol-gel matrix). The photosol-gel matrix was used for the first time as an organic dye molecule. Compared with the conventional sol-gel process, the photoinduced process offers several advantages: an increase in overall speeds linked to the sol-gel process allowing materials to be obtained in minutes or even seconds, the process does not use any solvent resulting in a reduction in emissions of polluting products, and the system does not need the addition of water but uses atmospheric humidity to initiate the sol-gel reactions. The photosol-gel inclusion the study dye (DBDMA) was prepared under 255 nm UV light (photoreactor) with an intensity of irradiation of 250 microwatts/cm^2^. Additionally, the fluorescence emission intensity of the DBDMA in the presence and absence of gold nanoparticles in different solid media will be measured. 

## 2. Materials and Methods

### 2.1. Materials

**Scheme 1:**
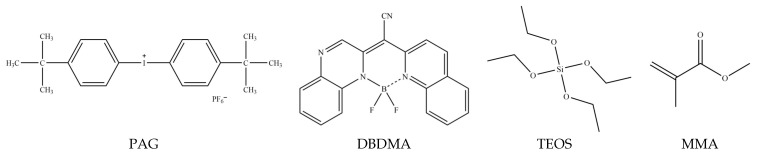
Chemical structures of monomers and additives used in this study.

The studied 2-(1-(difluoroboraneyl)-1,2-dihydroquinolin-2-yl)-2-(1 methylquinoxalin-2-ylidene) acetonitrile (DBDMA) dye is kindly supplied by Professor Ewald Daltrozzo of the Faculty of Chemistry in Konstanz University located in Konstanz, Germany. The silica precursors TEOS (Tetraethyl orthosilicate, purity > 99.99%), Absolute Ethanol (EtOH, ≥99.8%), Potassium gold (III) chloride, (KAuCl_4_, purity ≥ 98%), Bis(4-tert-butylphenyl) iodonium hexafluorophosphate (PAG), (purity ≥ 98%), Methyl methacrylate (MMA), and the reducing agent Sodium citrate (Na_3_C_6_H_5_O_7_, purity ≥ 99%), were all supplied by Sigma Aldrich (St. Louis, MO, USA) and used without further purification. Hydrochloric acid (HCl, 35 %W/W) was obtained from Merck (Darmstadt, Germany). The following Scheme 1 shows the structure of the chemical compounds used in the study.

### 2.2. Synthesis of Sol-Gel Hybrid Matrials 

#### 2.2.1. Preparation of Classical Sol-Gel Process 

Sol-gel matrix was prepared by the hydrolysis of silicon alkoxide then polycondensation. This method involves the addition of HCl as a catalyst and presentation of glycerol to prevent cracking of monoliths through drying. During synthesis, two parts of the solution were prepared. In the first part: 11.2 mL of (TEOS) was selected as the silica source mixed with 6 mL ethanol (EtOH). The solution was stirred for 30 min. The second part of the solution was then prepared by mixing 9 mL deionized water with an acidic medium from 1 mL HCl (0.1 N) alongside the addition of 8 mL glycerol. These two solutions were then mixed and stirred. At this stage, the solution is kept under stirring at room temperature for about 14.5 h [30]. By the doping method, the dye is incorporated into the sol-gel matrix by including the solution: DBDMA sample concentration (5.8 × 10^−5^ M, in ethanol) solution: sol-solution in the molar ratio 1 mL:2.5 mL and then drying in the oven at 60 °C. After approximately 21 days, the samples became dry, and it was possible to carry out the measurements [31]. It is worth mentioning that was put it in a glass cuvette (polystyrene) and then sealed with Teflon tape for three weeks to dry as the blank sample [16,17,18]. Likewise, the dye is incorporated into the sol-gel matrix in the presence of gold NPs prepared by sodium citrate (1 × 10^−2^ M) according to the following molar ratios: DBDMA sample concentration (5.8 × 10^−5^ M, in ethanol) solution: sol-solution: gold chloride solution (1 mL:2.5 mL:2 mL).

#### 2.2.2. Synthesis of Photosol-Gel and Organic–inorganic Photopolymerization

In the typical procedure, 2 wt% of Bis(4-tert-butylphenyl) iodonium hexafluorophosphate (PAG) was dissolved in inorganic precursors TEOS in both the presence and absence of 1 wt% of DBDMA. Other samples were prepared with gold chloride 4 wt%. The resulting solution was stable in the dark with a pot life exceeding 4 months. The solution was put into a Pyrex tube and irradiated at 255 nm in atmospheric air at room temperature. Experiments were carried out in an environmental cell where the temperature was controlled at around 23 ± 1 °C and the RH was maintained at around 21%. 

### 2.3. Characterization

UV-Vis electronic absorption spectra were recorded on UV-Vis Shimadzu 1650 Spectrophotometer (Shimadzu, Duisburg, Germany). The fluorescence properties of the DBDMA were determined using a JASCO FP-8200 spectrometer (JASCO, Riyadh, Saudi Arabia). 

The morphology and particle size of the polymer were examined by Field Emission Scanning Electron Microscope (FESEM) (JEOL JSM 6490-A) at different resolutions.

FTIR spectra have been obtained by Shimadzu FTIR spectrometer (Shimadzu (model 8000), Duisburg, Germany). The range of measuring ranged from 400 to 4000 cm^−1^. The sample was fixed on a holder of KBr. 

### 2.4. Irradiation Source

The samples were typically irradiated in a photoreactor consisting of ten UV lamps at 254 nm with an irradiation intensity of 250 microwatts/cm^2^ without a water-cooling system under atmospheric air at room temperature.

### 2.5. Formatting of Mathematical Components 

To estimate the fluorescence quantum yield $\emptyset_{f\;}$ of the DBDMA in sol-gel and photoinduced sol-gel matrices, rhodamine 6G in methanol solution was used as a reference with a value of quantum yield of 0.96 [32,33]. The following Equation (1) was applied to calculate the fluorescence quantum yields:

(1) $$\emptyset_{f}\left(s\right)=\emptyset_{f\;}\left(r\right)\times \frac{\int^{\;}I_{s}}{\int^{\;}I_{r}}\times \frac{A_{r}}{A_{s}}\times \frac{n_{s}^{2}}{n_{r}^{2}}$$

The integrals represent the corrected fluorescence peak areas, *A* is the absorbance at the excitation wavelength, and *n* is the refractive index of the solvent used. The subscripts *s* and *r* refer to sample and reference, respectively. 

The oscillator strength (*f*) and transition dipole moment from ground to excited state (*µ*_12_) are calculated using Equations (2) and (3) [34,35]. The oscillator strength is defined as the number of active electrons traveling from the ground to the excited state. The absorption cross-section (*σ*_a_) was calculated according to Equation (4) [8].

(2) $$f=4.32\times 10^{-9}\int^{\;}\varepsilon (\bar{v)}d\bar{v}$$

*ε* is the numerical value of the molar absorption coefficient measured in M^−1^ cm^−1^ and $\;\bar{v}$ is the wavenumber value measured in cm^−1^.

(3) $$\mu_{12}^{2}=\frac{f}{4.72\times 10^{-7}E_{max}}$$

*E*_max_ is the maximum absorption energy in cm^−1^ and (*f*) is the oscillator strength.

*σ*_a_ = 0.385 × 10^−20^ *ԑ*(*λ*) (4)

where *ε*(*λ*) is the molar extinction coefficient.

## 3. Results

### 3.1. Photophysical Proprieties of Sol-Gel and Photosol-Gel DBDMA Doping

#### 3.1.1. Absorption and Emission in Liquid and Photoinduced Sol-Gel Media

**Figure 1:**
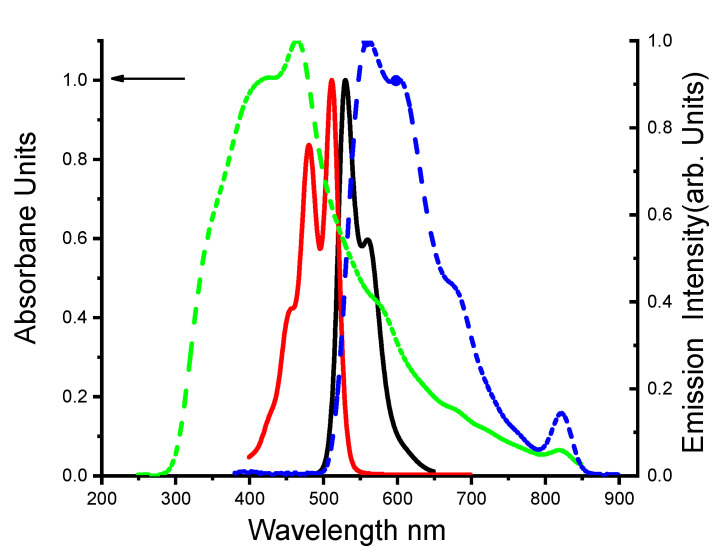
Normalized electronic absorption spectra and fluorescence spectra of 1.41 × 10^−5^ M DBDMA in dimethylformamide at 25 °C. The lines represent **ـــــــــ** absorption and **ــــــــ** fluorescence spectra in the solution state, and the dotted lines represent **ـ ـ ــ** absorption and **ـ ـ ـ** fluorescence spectra in photosol-gel media, respectively (λ_ex_ = at λ_ab max_).

The fluorescence and absorption spectra of DBDMA in dimethylformamide-liquid and the photoinduced sol-gel glasses are shown in Figure 1. The UV/Vis absorption and emission spectra of DBDMA were obtained at room temperature and measured at a concentration of 1.411 × 10^−5^ mol dm^−3^. 

The (DBDMA) has intense electronic absorption spectrum bands at 512 nm and 481 nm, with a high molar absorption coefficient (*ε*) (42,945.22 M^−1^ cm^−1^) and oscillator strength (*f*) in the range of 0.15, as the kind of solvent matter. The value of the dipole moment of the DBDMA molecule in DMF is 4.07. The last one is typical, indicating that the S_0_→S_1_ transition is a strongly permitted type π→π*. The weak absorption peak at approximately 481 nm indicates S_0_→S_2_ transitions [34,36]. The absorption spectrum has vibronic peaks, which lost in the emission spectrum, indicating that the planarity of the molecule is lost upon excitation, and exhibiting that the shoulder peaks at 450 nm correspond to the vibrational band of the S_0_–S_1_ transitions [36]. The fluorescence maximum of DBDMA was observed at 530 nm in liquid media (S_0_←S_1_), and a red-shift was obtained in fluorescence maximum (ca. 18 nm), indicating that the S_1_ of the DBDMA has higher polarity than the S_0_. This means that a change in the dipole moment of DBDMA molecule occurs when it is excited [34]. The DBDMA dopped in photosol-gel matrix was prepared and characterized (see Figure S3). This later, shows absorption maximum at 465 nm and strong red emission with peak maximum at 562 nm when it is excited at 465 nm. This behavior was attributed to the emission from the accumulation of the excited state with a fluorescence of 97 nm red-shifted relative to the emitted solid state (photosol-gel matrix) [34]. 

On the other side, a large stokes shift of around 97 nm was obtained for the DBDMA dopped in the photosol-gel media. This one usually ascribes to the excited state reactions, most often proton transfer. The red shift could be due to vibrational relaxation of the initially excited state to the lowest vibrational state of the first electronic excited state. Emission occurs to a variety of vibrational states in the ground state causing the red-shifted emission.

A high molar absorption coefficient of 77,817.15 M^−1^ cm^−1^ is obtained compared to the liquid media listed (see Table 1). The oscillator strength (*f*) in photosol-gel matrix and dipole moment values are shown also in Table 1.

#### 3.1.2. Absorption and Emission in Liquid Media and Classical Sol-Gel

**Figure 2:**
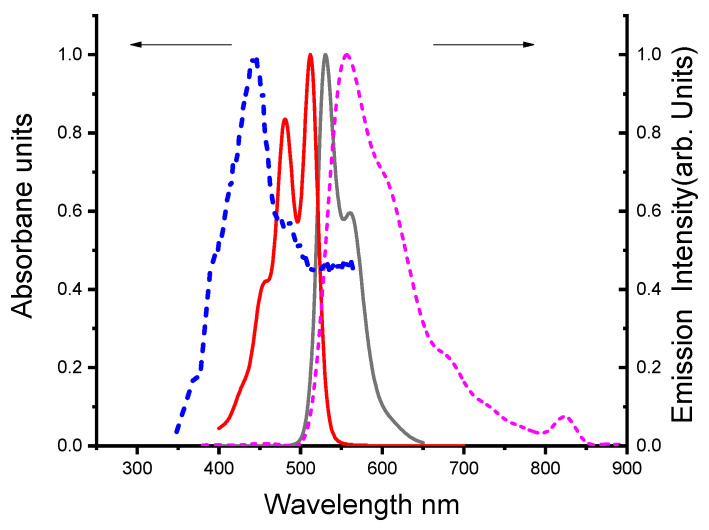
Normalized electronic absorption spectra and fluorescence spectra of 1.41 × 10^−5^ M DBDMA in DMF at 25 °C. The lines represent **ـــــــــ** absorption and **ــــــــ** fluorescence spectra in solution state, and the dotted lines represent **ـ ـ ــ** absorption and **ـ ـ ـ** fluorescence spectra classical sol-gel media, respectively (*λ*_ex_ = at *λ*_ab max_).

The synthesis and the characterization of classical sol-gel with the inclusion of DBDMA were presented in Supplementary Materials. Effective emission in both the solution (DMF) and the classical sol-gel is shown in Figure 2. Fluorescence spectra of the classical sol-gel give a red transformation emission range at 557 nm compared to its emission range at 530 nm in a solution, respectively by 27 nm. The absorption of DBDMA in liquid media at 512 nm is largely converted to blue in the solid-state by 67 nm at approximately 445 nm. The dye shows a stock shift (ca. 18 nm) between the absorption and emission spectra in liquid media. No significant red transformations were observed in the DMF solution, indicating the formation of neither aggregation in the ground state nor excimer in the excited state. In contrast, the absorption and emission spectra of classical sol-gel turn significantly red by 112 nm, due to the presence of interactions between the particles in classical sol-gel [15]. Apparently, incorporating the DBDMA into an inorganic sol-gel matrix (silica matrix) had an impact on the photochemistry of the dye molecules compared to the DMF solution. It is likely that due to the cationic character of the DBDMA during the hydrolysis step, the dye tends to be absorbed on the surface of the oxide network, so the dye properties are affected by the surface properties. As a result, short-term reactions that are responsible for changes in the behavior of the dye occur. In fact, the effect of pH on the formation of the gel plays a role in changing the kinetics of polymerization and the production of charge on the surface of the gel since at a high pH (pH = 4) the net charge of the silica polymer is expected to be negative, with SiO- groups on the surface of the gel particles. The (+DBDMA) form can be stabilized over a large pH range by electrostatic interaction with SiO- groups on the network surface [37]. Table 1 listed the value of the molar absorption coefficient 44,006.4 M^−1^ cm^−1^, the oscillator strength (*f*) in the sol-gel matrix, and the dipole moment. 

#### 3.1.3. Comparison of Absorption and Emission Spectra of the DBDMA in Different Solid Media

**Figure 3:**
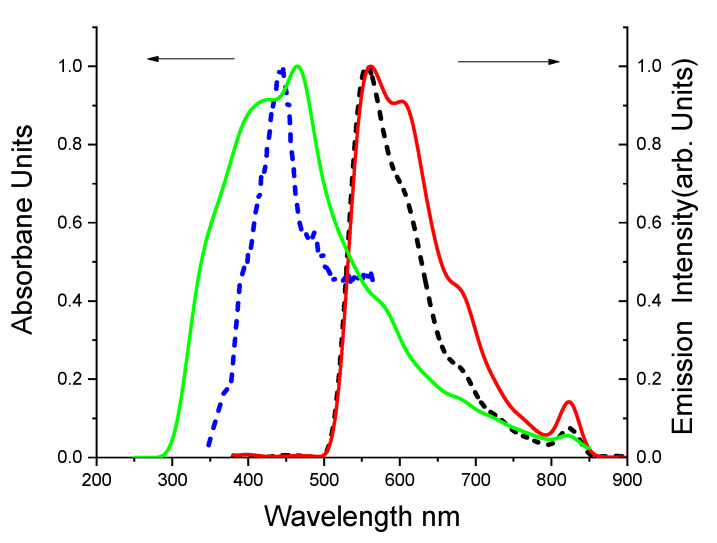
Normalized absorption and fluorescence spectra of 1.41 × 10^−5^ M DBDMA in DMF were described in solid media. The line 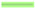 represents the absorption and 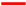 represents the fluorescence spectra in photosol-gel matrix, while **ـ ـ ـ** represents absorption and **ـ ـ ـ** represents fluorescence spectra in sol-gel matrix (λ_ex_ = at λ_ab max_).

Figure 3 displays the electronic absorption and fluorescence emission spectra of the DBDMA doped in different solid media (photosol-gel and classical sol-gel matrices). For DBDMA, the interference between the absorption and emission spectra in the sol-gel matrix is lower than photosol-gel matrix, which is beneficial to gain material efficiency as there will be less self-absorption of the Emission by the sample [38]. Thus, photon reabsorption of the DBDMA in the sol-gel matrix is minimal compared to a photosol-gel matrix. Referring to the maximum absorption in the conventional-gel matrix, it is shifted to a blue shift of about 20 nm towards a shorter wavelength compared to a photosol-gel matrix (see Figure 3) due to the disappearance of electronic transition (*n*-π*) in the proton of nitrogen heteroatoms [20] while the maximum emission wavelength is converted to a longer wavelength (redshift) in a photosol-gel and a sol-gel matrix from 557 nm to 562 nm respectively (ca. 5 nm). The oscillator strength value in photoinduced sol-gel (0.67) is higher than in sol-gel matrix (0.3). Hence, the effective number of electrons transferred from the ground to the excited states in sol-gel is lower than in photosol-gel. While the oscillator strength value in the solution state is less than that of the solid state (0.15), both the absorption cross section(σa) and oscillator strength values (*f*) were calculated [39] (details in Table 1). Also displayed in Table 1 is that the value of the dipole moment of the [DBDMA/photo sol-gel matrix] is higher compared to DBDMA molecule in DMF and [DBDMA/sol-gel matrix]. Indicating that the highest intramolecular charge transfer (ICT) is in the [DBDMA/photosol-gel matrix [32].

#### 3.1.4. The Emission of DBDMA in Different Solid Media in the Presence of Gold NPs

**Figure 4:**
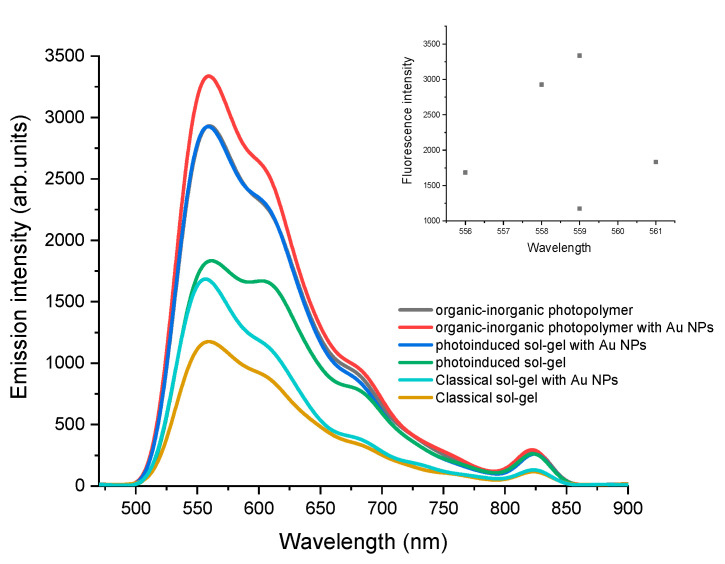
Comparison of 1.41 × 10^−5^ M DBDMA fluorescence intensity in different solid media with and without gold nanoparticles as the dye was dissolved in DMF (λ_ex_ = at λ_ab max_).

The fluorescence emission intensity of the DBDMA with and without gold nanoparticles in different solid media is described in Figure 4. We noticed that the DBDMA doping in organic–inorganic matrix (MMA-TEOS) is the best system. MMA was chosen as the pivotal component in the formula developed because of its excellent optical transparency and relatively high laser resistance [40]. The last system has a higher fluorescence intensity (I = 3336.5) compared to the other systems. The classic sol-gel matrix shows the lowest efficiency (low fluorescence intensity (I = 1175.4)). Therefore, these results revealed that DBDMA encapsulated in Au nanoparticles still maintained a strong emission intensity, and this indicated that the nanoparticles were transparent to the encapsulated dye. Moreover, the nanoparticles provide more protection for the dye which can increase emission intensity with lower dye concentration compared to DBDMA solution [27]. In the results of the current experiments, there are two specific mechanisms that are important to enhance the fluorescence intensity: the first one is the possibility of charge exchange with the dye and matrix, and the second one is that the presence of Au nanoparticles can cause a dipole-dipole interaction. Au NPs are known to contain a positive charge at the core and free negatively charged electrons on their surface [41]. On the other hand, the DBDMA is kept in the same polymer matrix with the only difference being in the presence or absence of the AuNPs. This is important because it indicates that the polymer matrix also had an effect on the fluorescence of the incorporated dye, suggesting that fluorescence quenching can occur by free-electron transfer from amino groups of DBDMA [42]. 

The presence of MMA with the photoinduced sol-gel formulation can decrease the polarity, resulting in a decrease in the relative amount of the silanol groups in the inner pore surface. This can explain the higher intensity observed in dye doping in MMA-TEOS matrices [43,44].

## 4. Conclusions

The following conclusions can be drawn from the above study:

i. During this study, classical sol-gel glasses with the inclusion of DBDMA dye were synthesized in both the presence and absence of Au-NPs (see Supplementary Materials). The hybrid materials were characterized by FTIR and SEM. The photosol-gel was also prepared as a matrix of DBDMA dye. The production of gold NPs occurred during the photosol-gel process at 255 nm. It was proved by UV-Vis characterization that the Au^+3^ was reduced by DBDMA to Au^0^ under irradiation at 255 nm and 250 mW/cm^2^.
ii. The reason for choosing the photosol-gel matrix was because of its high rigidity and transparency. On the other hand, the preparation of photosol-gel does not require the addition of water or solvents and is carried out in a single step.
iii. The optical properties such as *ε* (molar absorptivity), *f* (oscillator strength), μ_12_ (transition dipole moment), and φ*_f_* (fluorescence quantum yield) respectively for the target molecule were obtained in sol-gel, photosol-gel matrices and were compared to liquid media. It was noticed that the photosol-gel is the best matrix for the DBDMA dye. High fluorescence quantum yield of around 0.5 was obtained compared to the sol-gel matrix and the liquid media. The molar absorptivity was around 77,817.15 M^−1^ cm^−1^.
iv. The addition of gold NPs to the DBDMA molecule in different matrices enhances the fluorescence intensities. The best system obtained is DBDMA doping in (MMA-TEOS) matrix in the presence of gold nanoparticles. The high intensity obtained in the presence of MMA with the photosol-gel formulation may be due to the decrease in polarity, which leads to a decrease in the relative amount of silanol groups in the inner pore surface.

These obtained results indicated that the dye molecules DBDMA encapsulated in the photosol-gel matrix in the presence or absence Au-NPs had a high stability and retained their optical activity, thus providing a viable route for divers applications such as laser dye.

## Figures and Tables

**Table 1:** Photophysical parameters of DBDMA in different media.

	Liquid Media	Photosol-Gel	Classical Sol-Gel
*λ*_abs_ (nm)	512	465	445
*λ*_em_ (nm)	530	562	557
ε M ^−1^ cm^−1^	42,945.22	77,817.15	44,006.4
Φ*_f_*	0.34	0.5	0.4
*f*	0.15	0.67	0.3
*μ*_12_ *Debye*	4.07	8.83	5.8
*Stock shift* *(*nm*)*	18	97	112
*σ_a_* cm^2^/molec	1.7 × 10^−16^	2.99 × 10^−16^	1.69 × 10^−16^

## Data Availability

May be found from the cited references.

## Supplementary Materials

The following are available online at https://www.mdpi.com/article/10.3390/polym13162743/s1, Figure S1: In-situ time-resolved FT-IR spectra of silica hybrids TEOS containing (HCl, 0.1 N) as a catalyst, at 60 °C in the range of 4500 cm^−1^–400 cm^−1^. Figure S2: In-situ time-resolved FT-IR spectra of silica hybrids TEOS containing (HCl, 0.1 N) as a catalyst, and gold nanoparticles (10 mM) at 60 °C in the range of 4500 cm^−1^–400 cm^−1^. Scheme S1: Mechanism of UV-induced photolysis of a diaryliodonium salt (A), tetraethyl orthosilicate (TEOS) polymerized network formation (B). Figure S3. FTIR study of the photo sol-gel process of the sample TEOS/Dye/Φ_2_I^+^PF_6_^−^,  fresh and,  after 600 s of irradiation using photoreactor (λ_irr_ = 254 nm), intensity 250 mW/cm^2^. Figure S4: Evolution of the absorption spectra of the irradiation of 5.8 × 10^−5^ M DBDMA (λ_irr_ = 254 nm). Solution: 5.8 × 10^−5^ M DBDMA, 2 × 10^−6^ M gold chloride dissolved in DMF. Figure S5: (a) In-situ time-resolved FT-IR spectra of silica hybrids TEOS containing 2 wt% of PAG, DBDMA, and gold chloride 4 wt% under UV irradiation time 600 s in the range of 4500 cm^−1^–400 cm^−1^. (b) SEM image of silica hybrids TEOS containing 2 wt% of PAG, DBDMA, and gold chloride 4 wt% under UV irradiation time 600 s. Figure S6: (a) In-situ time-resolved FT-IR spectra of silica hybrids TEOS containing 2 wt% of PAG, MMA, DBDMA and gold chloride 4 wt% under UV irradiation time 600 s in the range of 4500 cm^−1^–400 cm^−1^, (b) SEM image of silica hybrids TEOS containing 2%wt of PAG, MMA, DBDMA and gold chloride 4 wt% under UV irradiation time 600 s.
