# Supplementary material for: Photoinduced and Classical Sol-Gel Synthesis: Spectral and Photophysical Behavior of Silica Matrix Doped by Novel Fluorescent Dye Based on Boron Difluoride Complex
Source: Polymers (Basel). 2021 Aug 16;13(16):2743. doi: 10.3390/polym13162743 (PMC8400351; doi:10.3390/polym13162743)
Supplement: Supplementary file 1 [file polymers-13-02743-s001.zip › polymers-1322086-supplementary.pdf]

polymers

MDPI

Supplementary Materials: Photoinduced and Classical Sol-Gel Synthesis: Spectral and Photophysical Behavior of Silica Matrix Doped by Novel Fluorescent Dye Based on Boron Difluoride Complex

1. Characterization of Sol-Gel and Photosol-Gel Materials

1.1. Classical sol-gel doped with DBDMA

In sol gel study different techniques were used: FTIR Spectroscopy, UV-vis. Spectrometer and Scanning Electron Microscope (SEM).

Figure S1 shows sequences of IR spectra, recorded as the hydrolysis and condensation proceeded, in the system TEOS–EtOH–H_2O–HCl (molar ratio of TEOS: EtOH: H_2O: HCl ~ 11: 6:9:1). In addition, one may observe a band at ~853 cm^{-1}, due to CH_3 or CH_2 deformation in EtOH, during the first one hour and 20 min of the sol-to-gel conversion, followed by a substantial reduction of its intensity afterward. The aqueous condensation, which begins while the hydrolysis is still in progress, will cause the removal of the ethanol, as well as the crosslinking of the oligomer. Absorption bands are observed 1032 cm^{-1} and near 923 cm^{-1}. A somewhat broader band replaces the relatively sharp band at ~923 cm^{-1} (rocking of CH_3 in TEOS) near the same position, characteristic of a wet gel (indicating the presence of Si–OH or Si–O– species) [1]. The formation of the siloxane band (Si-O-Si) after the condensation was observed at approximately 1032 cm^{-1} [1]. On the other hand, the region encompasses mainly the Si-OH (3272 cm^{-1}), due to the hydrolysis during the sol-gel reaction[2, 3]. Finally, the gel-glass conversion can be followed by the disappearance of the IR peaks due to the non-hydrolyzed alkoxy groups and to the Si—OH stretching, as well as the intensification of the peak at 853 cm^{-1} (See Fig.S1) [1].

In summary, the sol-gel evolution is based on the consumption of ethanol and TEOS and the formation of Si - O - Si bonds, which is demonstrated by the disappearance of the bands linked to ethanol and TEOS and by the simultaneous formation and intensification of bands bound to siloxane (Si - O - Si). The equation (1) presents the hydrolysis and the condensation reactions presented in (2 and 3):

Hydrolysis

$$\mathrm{Si(OR)_4 + 4\,H_2O \underset{esterification}{\overset{hydrolysis}{\rightleftharpoons}} HO\text{-}Si(OR)_3 + ROH} \tag{1}$$

Condensation

$$\mathrm{(RO)_3Si\text{-}OH + HO\text{-}Si(OR)_3 \underset{hydrolysis}{\overset{water\ condensation}{\rightleftharpoons}} (RO)_3Si\text{-}O\text{-}Si\text{-}(OR)_3 + H_2O} \tag{2}$$

or

$$\mathrm{(RO)_3Si\text{-}OH + RO\text{-}Si(OR)_3 \underset{alcoholysis}{\overset{alcohol\ condensation}{\rightleftharpoons}} (RO)_3Si\text{-}O\text{-}Si\text{-}(OR)_3 + ROH} \tag{3}$$

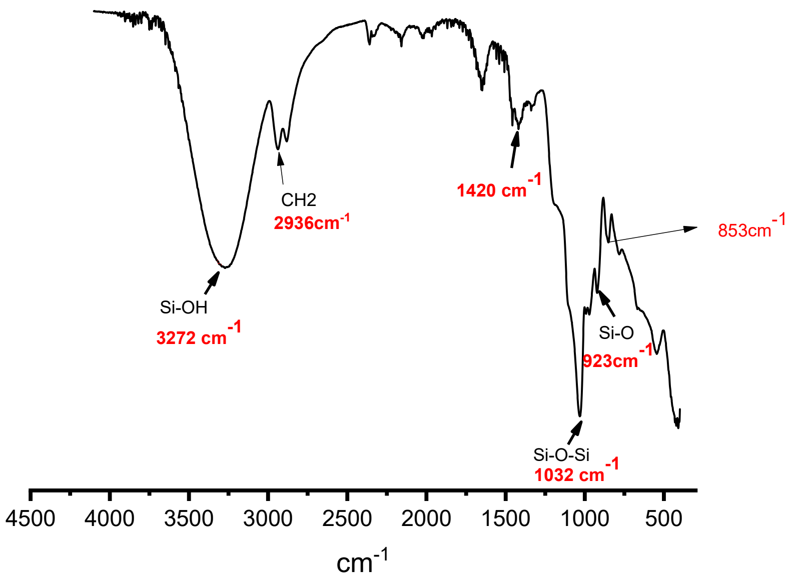

Figure S1. In-situ time resolved FT-IR spectra of silica hybrids TEOS containing (HCl,0.1N) as a catalyst, at 60°C in the range of 4500cm^{-1} – 400 cm^{-1}.

1.2 Classical sol-gel - Au-NPs doping with DBDMA

Figure S2 shows the Fourier transform infrared (FT-IR) spectrum of the DBDMA doping in the sol-gel matrix in the presence of gold nanoparticles. That was recorded in the frequency range of 4500 - 400 cm^{-1} to examine functional groups on the surface of the silica matrix during the stabilization of the metallic particles. FTIR spectra analysis showed intense bands at 2936.3, 1651.6, 1330,7, 1417,2, 1196,6, 600 cm^{-1} for the flower-shaped gold nanoparticles (See Fig.S2) [4]. FTIR spectrum of sol-gel-AuNPs solution shows a peak at 3385 cm^{-1} due to the binding of the hydroxyl group with gold nanoparticles [5]. The band at 2900–3000 cm^{-1} is due to C-H stretching modes of CH_3 group in TEOS, and the band at 3657 cm^{-1} corresponds to the vibrational stretching mode of surface silanol groups and water [6]. The Peak at 2793 cm^{-1} is attributed to Si-OH bonding. Peaks at 1103, 880, and 433 cm-1 correspond to flexural vibration modes, asymmetric and symmetric stretching vibration modes of the Si-O-Si bond, respectively [6]. We also note the presence of vibrations that related to the asymmetric vibrations of Si-O-Si and the Si-O-Si bending mode; respectively they appeared at 1049 cm^{-1}, 792 cm^{-1} while 948 cm^{-1} correspond to the vibrations of the Si-OH bonding [6].It has been reported that upon the synthesis of gold nanoparticles in a reaction mixture, the color turns to ruby red or deep purple due to the surface plasmon resonance of gold nanoparticles. The reaction mixture containing: DBDMA: TEOS: EtOH: H_2O: HCl changed from yellow to deep purple, indicating that the sol-gel had the capacity to reduce $HAuCl4•3H_2O$, resulting in the formation of gold nanoparticles.

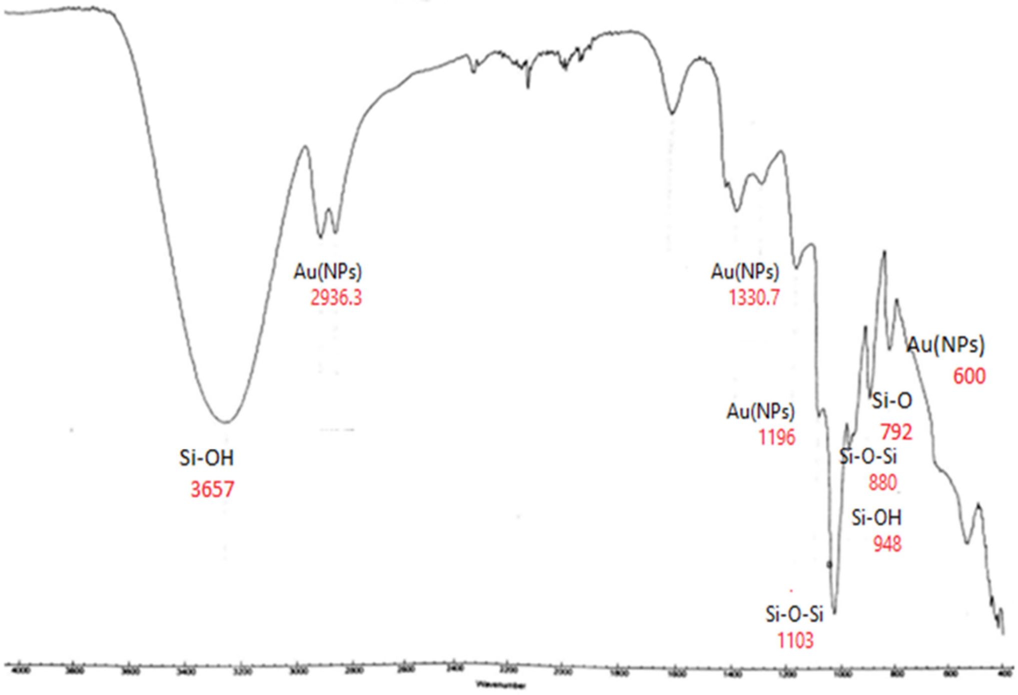

Figure S2. In-situ time resolved FT-IR spectra of silica hybrids TEOS containing (HCl,0.1N) as a catalyst , and gold nanoparticles (10 mM) at 60°C in the range of 4500cm^{-1} – 400 cm^{-1}.

1.3. photosol-gel doping with DBDMA

The sample containing TEOS, 2wt% of PAG, and 2wt% of the DBDMA is represented in Figure S3 after and before the exposure to UV irradiation. The irradiation time was 600 seconds. These spectra of these samples were also consistent with the development of a high degree of condensation for the silicate network. Complete hydrolysis of ethoxy group 950 cm^{-1} was observed through the disappearance of the C_2H_5–O symmetric stretch band. Despite the high level of condensation, large bands at 3458 cm-1 (hydrogen bonded OH stretching mode) and (Si–OH stretching mode) were still visible [1,7]. The Si–O–C_2H_5 group absorbing close to 2954 cm^{-1} (CH_3 symmetric stretch) strongly diminished after irradiation [8,9]. The asymmetric stretching band of siloxanes (Si–O–Si) at 1079 cm^{-1} and 1106 cm^{-1} also grew in a pronounced way, which is characteristic of silica network formation [9]. In the specific case of a photo sol–gel process, the extent of hydrolysis is mainly controlled by the ability of atmospheric water to diffuse within the sample (for this reason, the relative humidity was controlled and kept constant around 23%). The following scheme shows the acid generation during the UV irradiation of iodonium salt and then the formation of polymerized network.

A

B

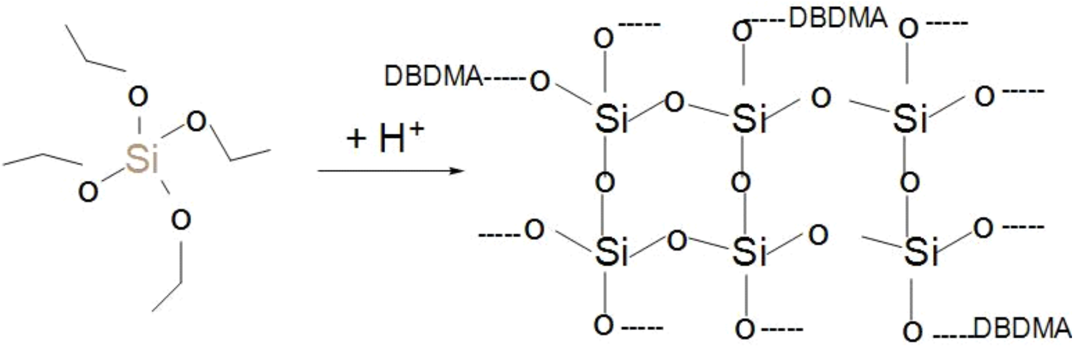

Scheme 1. Mechanism of UV-induced photolysis of a diaryliodonium salt (A), tetraethyl orthosilicate (TEOS) polymerized network formation (B).

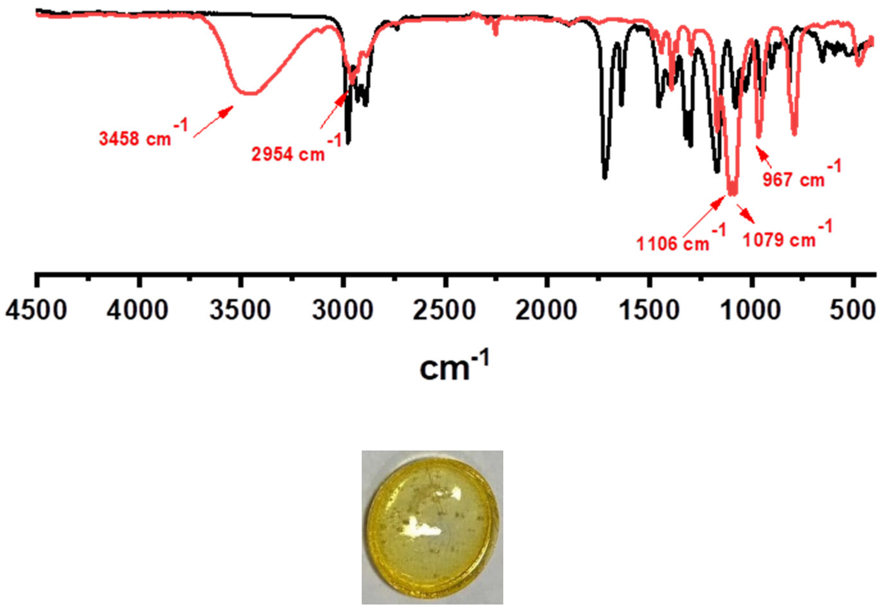

Figure S3. FTIR study of the photo sol–gel process of the sample TEOS/Dye/ $\Phi_2I^+PF_6^-$, fresh and, after 600s of irradiation using by photoreactor (λ_{irr}=254nm), intensity 250mW/cm^2.

1.4. Photosol-gel doping with DBDMA and gold nanoparticles

Photochemical production of gold NPs with DBDMA

For the synthesis of gold nanoparticles 2 ml of DBDMA concentration (5.8×10^{-5}M) was added to 1ml of gold chloride solution in DMF (2×10^{-6}M). The sample was stirred continuously for 5 min at room temperature. Figure S4 shows at t = 0s the appearance of the absorption band of Au^{+3} at 325 nm [10]. After 10 seconds of irradiation, we have seen a large decrease in the band at 325 nm and the change of the color to purple. After passing 80 seconds of irradiation, the absorption of the Au^{+3} has entirely disappeared; we notice the increase in absorption at 560 nm, which corresponds to the surface plasmon resonance (SPR) absorption. A careful analysis of the absorption spectrum during the irradiation period can be concluded that the cyanide ion oxidizes the metallic gold from Au^{+3} to Au^{+2}. The Au^{+2} is unstable and can be reduced with the cyanide ion to Au^{+1}. Then Au^{+1} can be reduced by another excited DBDMA to Au^0 [11-13].

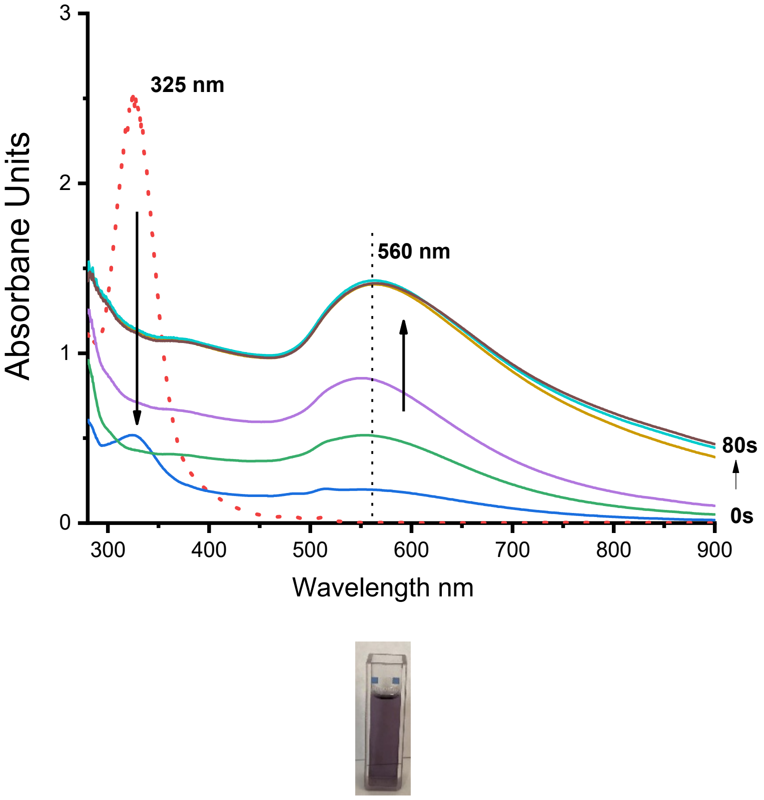

Figure S4. Evolution of the absorption spectra of the irradiation of 5.8×10^{-5}M DBDMA (λ_{irr}=254nm). Solution: 5.8×10^{-5}M DBDMA, 2×10^{-6}M gold chloride dissolved in DMF.

The FTIR is used to detect the functional groups and study the vibrational motion of atoms or molecules. In the case of photosol-gel doped dye in the presence of gold nanoparticles, we observed the existence of the bands at 2951 and 1588, and 673 cm-1. The peaks that confirm the formation of the sol-gel matrix were also observed in Figure 5S (a); it was mentioned in the previous explanations [4,8,9]. FT-IR spectra analysis showed intense bands at 3375, 2338, 1588, 1105, 7639, and 558 cm−1forthe flower-shaped gold nanoparticles (see Fig S5 (a)). The strong similar-it between the Classical sol-gel - Au-NPs mix and flower shape gold nanoparticles showed that the same photo sol-gel - Au-NPs were present in both.

The SEM images are presented in Figure S5(b). As seen, all the coatings had tight structures without comparable defects and cracks. Gold nanoparticles were dispersed uniformly in the polymer matrix, with a size of about 10-50 nm. Regarding the presence of the gold nanoparticles, visible in the picture as bright points, it can be noticed that they are approximately spherical in shape and randomly dispersed in the dielectric matrix. The SEM results show that the gold nanoparticles are distributed throughout the silicate matrix.

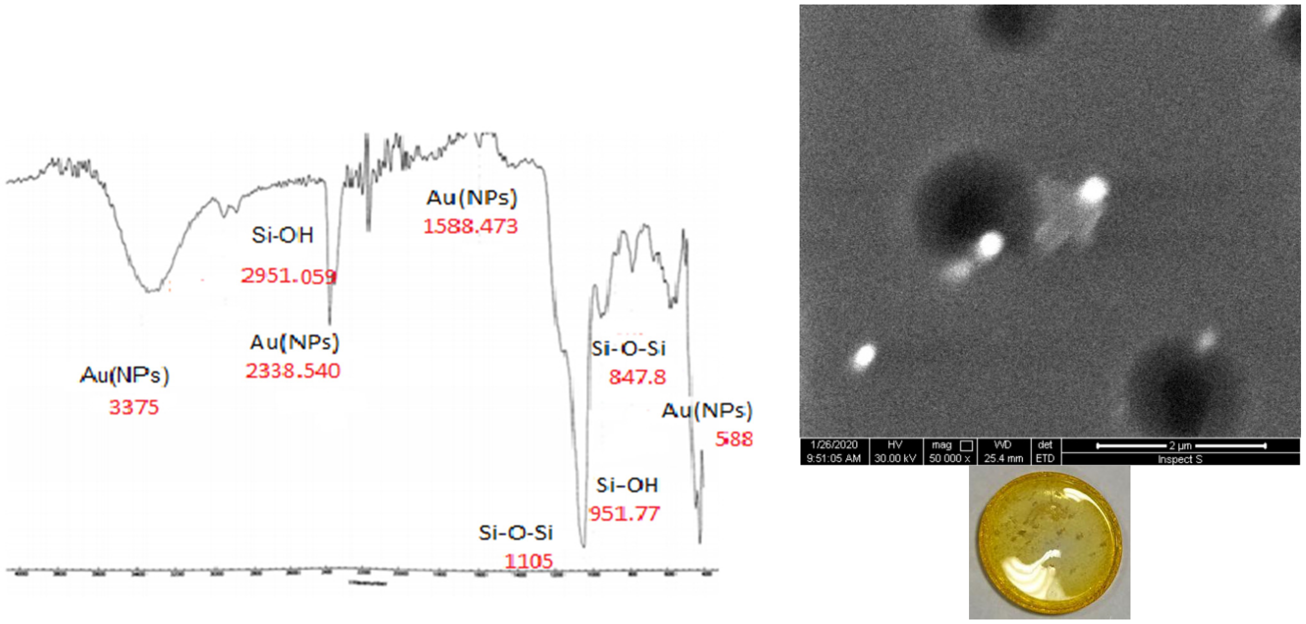

(**a**) (**b**)

Figure S5. **(a)** In-situ time resolved FT-IR spectra of silica hybrids TEOS containing 2%wt of PAG, DBDMA and gold chloride 4 wt% under UV irradiation time 600s in the range of 4500cm^{-1} – 400 cm^{-1}.(b) SEM image of silica hybrids TEOS containing 2%wt of PAG, DBDMA and gold chloride 4 wt% under UV irradiation time 600s.

3.1.5.Preparation of organic-inorganic hybrids in-situ gold NPs

Photosensitive organic-inorganic compounds appear as an alternative for the design of new optical devices because they combine the characteristics of glasses and photopolymers and improve the properties of the final material. This hybrid material exhibits a photo-induced reduction in the refractive index as well as a large volume contraction upon irradiation with ultraviolet (UV) light (190 - 280 nm).

Our study presents organic-inorganic material in-situ gold nanoparticles in the presence of DBDMA. The disappearance of the band at ν (C = C) 1627 cm^{-1} due to the consumption of the C = C double bond by the photoinduced process (see Fig. 6S (a)) [14]. The first region mainly includes the OH (3400 cm^{-1}) that involving weak and broad absorptions, corresponds with chemisorbed water [15]. Siloxane (Si - O-Si) bond can be seen in the range of 1000–1150 cm^{-1}. After the complete reaction, the peak of the Si - O-Si network structure appears at 1053 cm-1[14], as well as at approximately799 cm^{-1} appeared [2].

The scanning electron micrograph (SEM) shown in Figure 6S (b) reveals that the images were acquired using different conditions in order to highlight both the nanoparticle spreading and the surface morphology. The morphology of gold nanoparticles embedded in silica sol–gel matrix was examined, and it shows that the gold nanoparticles are distributed throughout the silicate matrix.

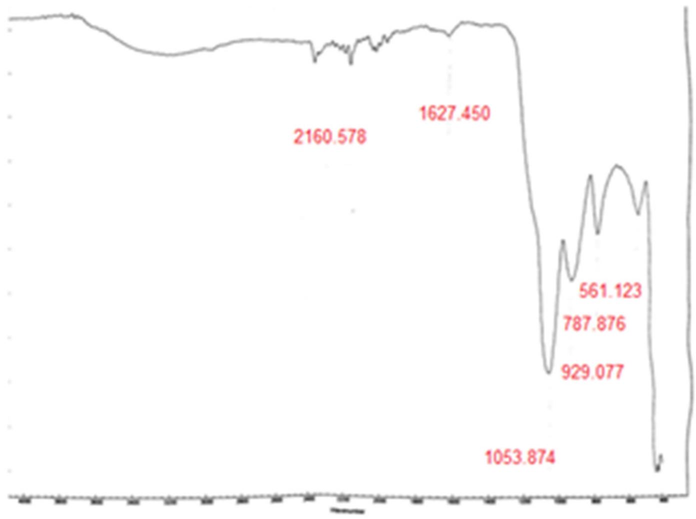

(**a**)

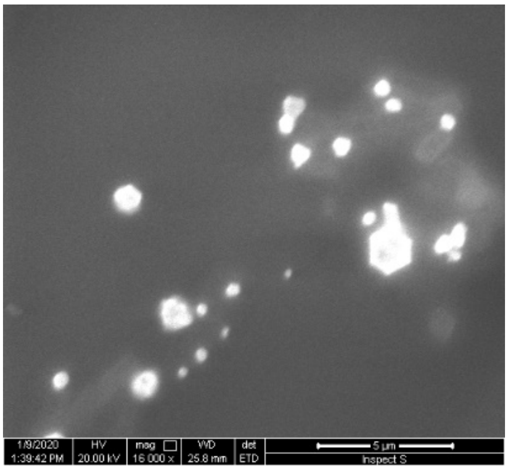

(**b**)

Figure S6. **(a)** In-situ time resolved FT-IR spectra of silica hybrids TEOS containing 2%wt of PAG, MMA, DBDMA and gold chloride 4% wt under UV irradiation time 600s in the range of 4500cm^{-1} – 400 cm^{-1} **, (b)** SEM image of silica hybrids TEOS containing 2%wt of PAG, MMA, DBDMA and gold chloride 4% wt under UV irradiation time 600s.

References

1. Ribeiro, T. V.; Santos, L. F.; Gonçalves, M. C.; Almeida, R. M. Heavily Yb-doped silicate glass thick films. *J. Sol Gel Sci. Technol.* **2017**, *81*, 105-113.
2. Pawlik, N.; Szpikowska-Sroka, B.; Pisarska, J.; Goryczka, T.; Pisarski, W. A. Reddish-Orange Luminescence from BaF2: Eu3 Fluoride Nanocrystals Dispersed in Sol-Gel Materials. *Materials* **2019**, *12*, 3735.
3. Kim, D.; Nam, S. Characterization of sulfonated silica nanocomposite electrolyte membranes for fuel cell. *Journal of nanoscience and nanotechnology* **2014**, *14*, 8961-8963.
4. Arundoss, T.; Arulkumar, S.; Senthilkumar, K.; Sabesan, M.; Vasudevan, K. ARTICLE INFO ABSTRACT. *Asian Journal of Science and Technology* **2013**, *4*, 140-144.
5. Tshabalala, M. A.; Gangstad, J. E. Accelerated weathering of wood surfaces coated with multifunctional alkoxysilanes by sol-gel deposition. *J. Coatings Technol.* **2003**, *75*, 37-43.
6. Czarnobaj, K. Preparation and characterization of silica xerogels as carriers for drugs. *Drug Deliv.* **2008**, *15*, 485-492.
7. Yanagida, S.; Nakagawa, T.; Kishi, T.; Yasumori, A. Preparation and characterization of gold nanoparticle-loaded silica-gel films for localized surface plasmon resonance sensing. *J. Sol Gel Sci. Technol.* **2015**, *74*, 227-233.
8. De Paz, H.; Chemtob, A.; Croutxé-Barghorn, C.; Le Nouen, D.; Rigolet, S. Insights into photoinduced sol–gel polymerization: an in situ infrared spectroscopy study. *The Journal of Physical Chemistry B* **2012**, *116*, 5260-5268.
9. Chemtob, A.; Belon, C.; Croutxé-Barghorn, C.; Brendlé, J.; Soulard, M.; Rigolet, S.; Le Houérou, V.; Gauthier, C. Bridged polysilsesquioxane films via photoinduced sol–gel chemistry. *New journal of chemistry* **2010**, *34*, 1068-1072.
10. Sonawane, R. S.; Dongare, M. K. Sol–gel synthesis of Au/TiO2 thin films for photocatalytic degradation of phenol in sunlight. *Journal of Molecular Catalysis A: Chemical* **2006**, *243*, 68-76.

11. de la Torre, E.; Gámez, S.; Pazmiño, E. In *Improvements to the cyanidation process for precious metal recovery from WPCBs;* Waste Electrical and Electronic Equipment Recycling; Elsevier: 2018; pp 115-137.
12. Scaiano, J. C.; Billone, P.; Gonzalez, C. M.; Marett, L.; Marin, M. L.; McGilvray, K. L.; Yuan, N. Photochemical routes to silver and gold nanoparticles. *Pure and Applied Chemistry* **2009**, *81*, 635-647.
13. Shah, A.; Qureshi, R.; Khan, S. B.; Asiri, A. M.; Shah, A. A.; Ishaq, M.; Khan, M. S.; Lunsford, S. K.; Zia, M. A. Spectroscopic analysis of Au-Cu alloy nanoparticles of various compositions synthesized by a chemical reduction method. *Advances in Materials Science and Engineering* **2015**, *2015*.
14. Kwak, S.; Kim, N. R.; Lee, K.; Yi, J.; Kim, J. H.; Bae, B. Enhancement of fluorescence and lasing properties of covalent bridged fluorescent dye in organic–inorganic hybrid materials. *J. Sol Gel Sci. Technol.* **2011**, *60*, 137-143.
15. Wu, H.; Tanabe, K.; Nagai, H.; Sato, M. Photo-Induced Super-hydrophilic Thin Films on Quartz Glass by UV Irradiation of Precursor Films Involving a Ti (IV) Complex at Room Temperature. *Materials* **2019**, *12*, 348.
